# Movements of Diadromous Fish in Large Unregulated Tropical Rivers Inferred from Geochemical Tracers

**DOI:** 10.1371/journal.pone.0018351

**Published:** 2011-04-06

**Authors:** Benjamin D. Walther, Tim Dempster, Mike Letnic, Malcolm T. McCulloch

**Affiliations:** 1 Australian Research Council Centre of Excellence for Coral Reef Studies, Research School of Earth Sciences, Australian National University, Canberra, Australian Capital Territory, Australia; 2 Marine Science Institute, The University of Texas at Austin, Port Aransas, Texas, United States of America; 3 Department of Zoology, University of Melbourne, Melbourne, Victoria, Australia; 4 School of Natural Sciences, University of Western Sydney, Penrith South DC, New South Wales, Australia; 5 Australian Research Council Centre of Excellence for Coral Reef Studies, School of Earth and Environment, The University of Western Australia, Crawley, Western Australia, Australia; University of Glamorgan, United Kingdom

## Abstract

Patterns of migration and habitat use in diadromous fishes can be highly variable among individuals. Most investigations into diadromous movement patterns have been restricted to populations in regulated rivers, and little information exists for those in unregulated catchments. We quantified movements of migratory barramundi *Lates calcarifer* (Bloch) in two large unregulated rivers in northern Australia using both elemental (Sr/Ba) and isotope (^87^Sr/^86^Sr) ratios in aragonitic ear stones, or otoliths. Chemical life history profiles indicated significant individual variation in habitat use, particularly among chemically distinct freshwater habitats within a catchment. A global zoning algorithm was used to quantify distinct changes in chemical signatures across profiles. This algorithm identified between 2 and 6 distinct chemical habitats in individual profiles, indicating variable movement among habitats. Profiles of ^87^Sr/^86^Sr ratios were notably distinct among individuals, with highly radiogenic values recorded in some otoliths. This variation suggested that fish made full use of habitats across the entire catchment basin. Our results show that unrestricted movement among freshwater habitats is an important component of diadromous life histories for populations in unregulated systems.

## Introduction

Diadromous fishes often travel extensive distances between their spawning and maturation habitats. By definition, these movements traverse salinity gradients at some life history stage, theoretically in order to continually maximize growth and survival despite ontogenetic shifts in risks and requirements [1]–[2]. While some diadromous species adhere to strict schedules of ontogenetic habitat shifts, others display a portfolio of tactics within a species or population, often including individuals that never cross major salinity gradients during their lifetime. This flexible portfolio of life histories may confer resilience in the face of unpredictable disturbances that render a given pattern of habitat use unsustainable for a period of time [3]–[4]. Diversity in habitat use patterns therefore represents a bet-hedging strategy, which may be of critical importance in the face of anthropogenic activities that increase the frequency, duration or intensity of disturbances. Resilience to disturbances is particularly important for diadromous species, which face a wide range of anthropogenic threats that have contributed to worldwide population declines [5]–[7].

Elucidating variation in diadromous life history strategies has been aided by the advent of high precision chemical analytical techniques that probe compositional variation across aragonitic fish ear stones, or otoliths [8]. Because otoliths are metabolically inert after formation [9], grow through the sequential accretion of layers [10], and incorporate certain elements in proportion to their ambient abundance [11]–[12], movements through chemically distinct bodies of water can be effectively reconstructed. Otolith chemistry has been particularly valuable for identifying the presence and timing of movements across substantial salinity gradients in diadromous fishes [13]–[15]. However, determining movements among habitats within fresh water using otolith chemistry has proved more challenging. This is principally due to reduced chemical gradients within tributaries compared to those found between fresh and marine biomes. However, when significant freshwater chemical gradients exist due to geological heterogeneity, movements between particular freshwater habitats can be retrospectively identified [16]–[18].

A variety of chemical tracers in otoliths have been used to reconstruct movements across salinity regimes. For example, gradients in ^87^Sr/^86^Sr can identify residence in fresh or marine habitats [17], [19]–[21]. The utility of these isotope ratios derives from the range of geologically-determined freshwater endmembers. Depending on streambed geological composition, these endmembers can vary significantly between or even within river systems [16], [22]. In addition, many freshwater endmembers are measurably distinct from the globally homogenous marine value of 0.7092 [23]. Because there is no significant trophic fractionation in ^87^Sr/^86^Sr ratios [24] (which in our study is corrected via the normalization to ^86^Sr/^88^Sr) and otoliths faithfully record ambient environmental ratios [17], [25], this tracer is an unambiguous marker of diadromous movements. Unfortunately, mixing curves between fresh and marine endmembers tend to flatten out at salinities of 5 to 15, rendering movements between mesohaline and fully marine habitats analytically indistinguishable [20]. Additional tracers are required to identify residence in particular salinity regimes.

Dissolved trace elemental ratios such as Sr/Ca and Ba/Ca generally differ in fresh and marine habitats. Ba derives primarily from terrigenous sources and dissolved concentrations are typically highest at low to moderate salinities due to desorption from particles in estuarine mixing zones [26]–[27]. Marine surface waters are depleted in Ba due to its nutrient-like distribution in the water column [28]–[29]. High ambient Ba is therefore indicative of estuarine or fresh water masses. In contrast, Sr is typically elevated in marine habitats compared to freshwater, although this relationship depends strongly on the freshwater endmember of the tributary, which is in turn determined by the surficial and bedrock geology [30]–[31]. For most systems, though, high ambient Sr usually indicates marine water masses. The contrasting relationships of these two elements with salinity make their combined use a potentially powerful way to discriminate movement across a range of salinities. McCulloch et al. [21] combined ^87^Sr/^86^Sr and Sr/Ba ratios to estimate habitat use patterns of barramundi *Lates calcarifer* (Bloch) in eastern Queensland to identify individuals that moved between fresh and marine regions and those that resided in transitional estuarine habitats. Extending this approach to elucidate use of freshwater habitats may provide further insight into the ecology of diadromous fish.

Barramundi are large and long-lived catadromous latid fish widely distributed across the Indo-West Pacific, including northern Australia. Due to its recreational and commercial importance, much work has focused on distribution patterns, large and fine-scale movements and habitat preferences [32]. Chemical compositions of scales [33]–[34] and otoliths [20], [35] have been used to detect spawning frequency and identify individuals that spend variable amounts of time in marine habitats prior to migration into fresh water. Extending this approach to elucidate the use of freshwater habitats may provide further insight into the ecology of diadromous fishes.

To test whether it is possible to distinguish between the different freshwater habitats used by barramundi in large, un-regulated rivers in northern Australia, we combined measurements of elemental (Sr/Ba) and isotope (^87^Sr/^86^Sr) ratios in otoliths. These large river systems include geologically heterogeneous regions, suggesting that movements among regions within rivers may be chemically detectable. The seasonally intermittent nature of streamflow in the Victoria River meant that we could collect fish with known residence periods in disconnected water holes, thus allowing us to determine spatial variation in ^87^Sr/^86^Sr and Sr/Ba ratios by analyzing otolith edge regions. Our specific aim was to test the following hypothesis: does otolith ^87^Sr/^86^Sr and Sr/Ba vary sufficiently among river sections to indicate intra-riverine movements?

## Methods

### Study systems

**Figure 1:**
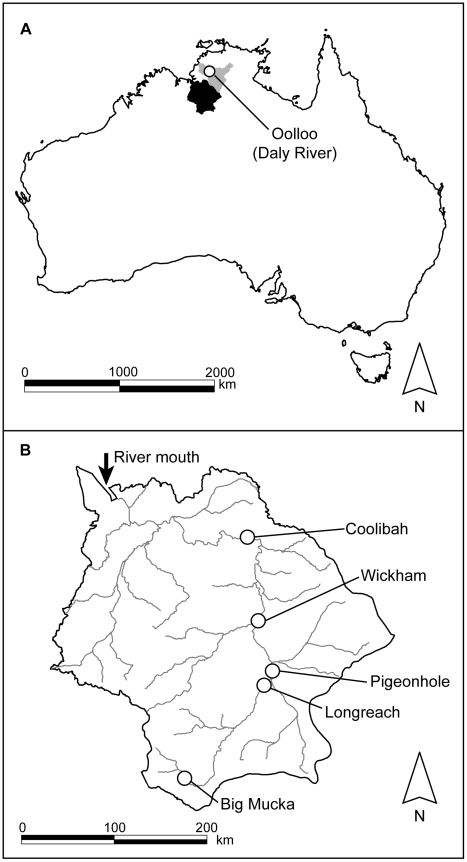
Map of river systems and sampling locations. Map of fish collection locations in the Northern Territory of Australia. (a) Watershed regions for the Victoria (black) and Daly (gray) rivers. The single sampling location in the Daly River is indicated. (b) Victoria River catchment with sample locations indicated.

We collected barramundi from two large unregulated rivers in the Northern Territory (NT) of Australia (Figure 1). The Daly River is the third largest river system in NT with a catchment size of approximately 53,000 km^2^ and a length of over 560 km from the mouth to its headwaters. Due to continuous discharge from three underlying aquifers, the mainstem of the Daly River is perennially free-flowing and has the highest base flow of all NT rivers [36]. The Victoria River is the longest NT river (over 700 km from mouth to headwaters) and has a large catchment size of nearly 88,000 km^2^. Flow in the Victoria River mainstem is highly seasonal. During the dry season (April/May to November) minimal precipitation and evaporative loss reduces baseflow until water remains only in isolated waterholes. Waterholes are isolated for between 3–7 months, depending on their location, before the onset of monsoonal precipitation restores habitat connectivity.

**Figure 2:**
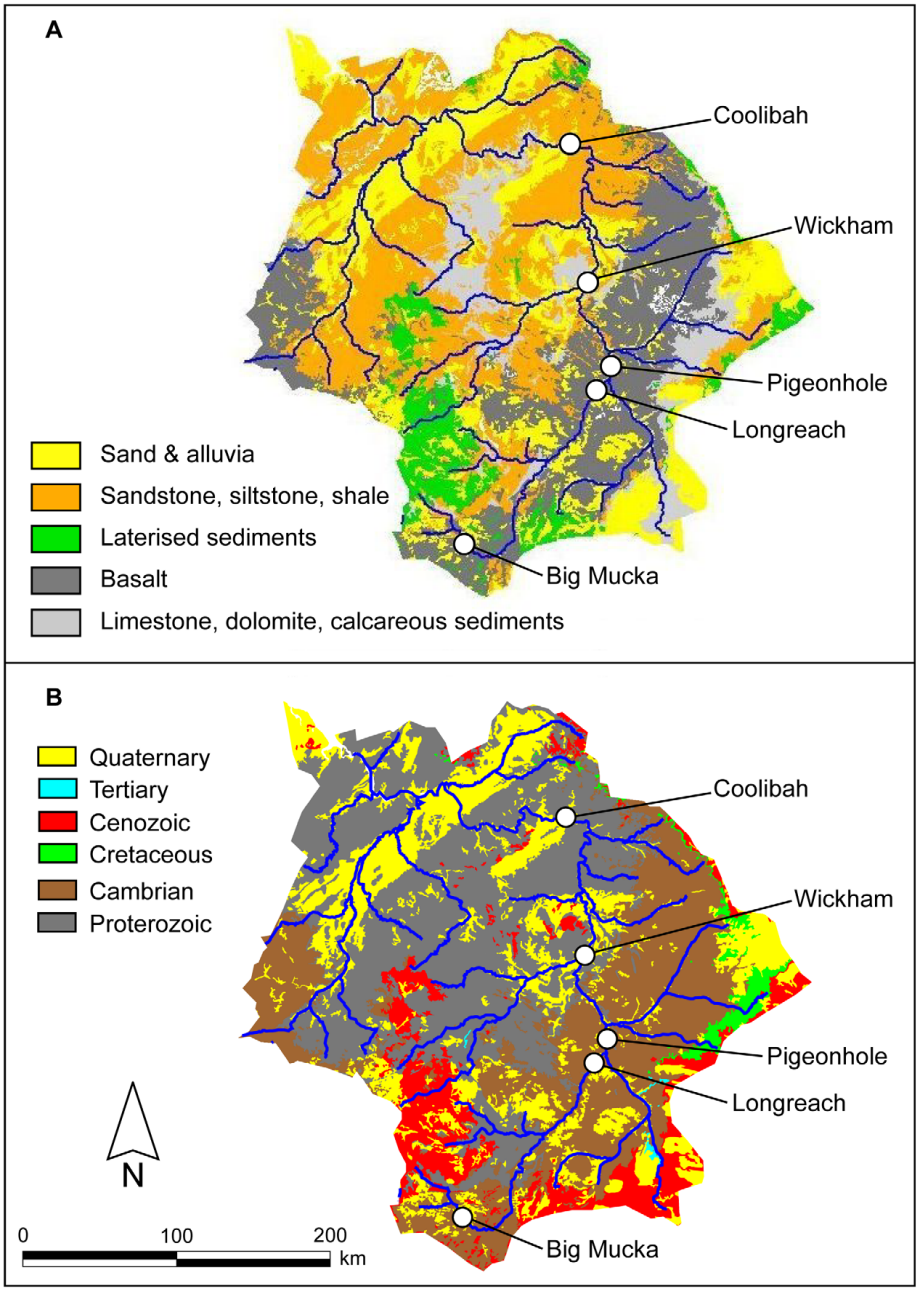
Map of Victoria River catchment geology. Catchment geology of the Victoria River. Map displays (a) streambed geological composition and (b) geological ages. Sampling locations are indicated by circles.

Streambed geology of the river systems is highly diverse (Figure 2). The Victoria River catchment contains a mix of unconsolidated alluvia, sandstones, shales, limestones, laterised sediments, and basalts. Geological ages are generally ancient for the majority of rock types, including large regions of Proterozoic and Cambrian formations. This heterogeneity of rock types and ages should therefore be reflected in a diversity of dissolved elemental and isotopic compositions across the catchment, particularly between the calcareous and basaltic regions.

### Fish collections

Barramundi were collected in October and November of 2008 at the end of the dry season prior to significant rainfall in the region. Fish were sampled from five separate waterholes along the Victoria River (Figure 1), where they had been resident for a minimum of 3–7 months to due waterhole isolation (Table 1). Fish were also collected from one location, Oolloo, in the Daly River, although perennial flow meant fish were not necessarily resident at the collection location for any known length of time. Fish were sampled using hook and line, measured (total length) and dissected for otoliths. Otoliths were rinsed and stored dry prior to analyses.

**Table 1:** Waterhole collection locations, river distance from the mouth, months of waterhole isolation prior to sampling, sample sizes and mean total lengths (cm) for analyzed fish.

River	Location	Distance upstream (km)	Months isolated	n	TL (SE)
Victoria	Big Mucka	578	7	4	66 (2)
Victoria	Longreach	424	6	7	62 (2)
Victoria	Pigeonhole	406	6	5	60 (1)
Victoria	Wickham	339	5	5	50 (4)
Victoria	Coolibah	221	3	8	55 (7)
Daly	Oolloo	277	0	6	44 (5)

### Otolith analyses

Otoliths were embedded in epoxy and sectioned through the transverse midplane using a low speed diamond wafering saw. Sectioned otoliths were then polished and mounted on petrographic slides for analysis. Otoliths were analyzed on both single and multiple collector inductively coupled plasma mass spectrometers (ICP-MS) in sequence, each coupled to a 193 nm ArF excimer laser. Prior to measurement on both instruments, analysis tracks were preablated to remove approximately 5 µm from the surface and exposing material free from contaminants. The laser was operated using an 80 µm spot size, a 5 hz repetition rate, a laser energy of 50 mJ with a 50% partially reflecting mirror, and a scan speed of 6 µm.s^−1^.

Otolith transects, hereafter referred to as life history profiles, were ablated from the core to the distal edge across the ventral lobe. Elemental (Sr/Ca, Ba/Ca) ratios were quantified with a Varian 820 ICP-MS by continuously monitoring ^43^Ca, ^86^Sr and ^138^Ba. Analyses were done in blocks of four to six otoliths bracketed by NIST glass standards 612 and 610. Background counts of monitored isotopes were collected for sixty seconds before and after each block for offline subtraction of background intensities. Elemental intensities were normalized to Ca and corrected for elemental bias using interpolations of NIST612 following Sinclair et al. [37] to obtain molar ratios. External precisions based on repeated measurements of NIST 610 over all analyses (*n* = 14) were 2.3% for Sr/Ca and 1.2% for Ba/Ca. Because Sr/Ca and Ba/Ca typically vary inversely across salinity gradients, ratios of Sr/Ba (ppm) were calculated to maximize detection of movement through mesohaline habitats.

After elemental ratio analyses, parallel tracks were ablated on each otolith to quantify ^87^Sr/^86^Sr ratios with a Thermo Neptune multi-collector ICP-MS. Analytical methods followed those described by McCulloch et al. [21] with the following modifications. Briefly, the masses ^83^Kr, ^84^Sr, ^85^Rb, ^86^Sr, ^87^Sr and ^88^Sr along with the mass positions 82.5, 83.5 and 86.5 were measured continuously and simultaneously with the nine-cup multi-collector array. Direct isobaric interferences of ^87^Rb on ^87^Sr and krypton isotopes on ^84^Sr and ^86^Sr were corrected for using the directly measured value of ^85^Rb/^87^Rb = 2.463. The krypton interference was corrected using a blank subtraction procedure with on-peak baselines measured before each sample. A *Tridacna* shell sample was measured between each sample to assess the validity of the interference correction procedure by monitoring ^84^Sr/^86^Sr ratios, which are most sensitive to Kr interference. The mean measured and corrected ^84^Sr/^86^Sr ratio of the *Tridacna* standard was 0.0566±0.0001 (SD) and within one standard deviation of the accepted value (0.0565). The mean measured and corrected ^87^Sr/^86^Sr ratio (normalized to ^86^Sr/^88^Sr = 0.1194) was 0.70916±0.00006 (SD), within one standard deviation of the global marine value (0.70918).

### Data analysis

Chemical analyses based on Sr/Ba and ^87^Sr/^86^Sr ratios enabled life history profiles to be derived for individual fish. Because the sampling frequency for ablations of ^87^Sr/^86^Sr ratios was lower than that of Sr/Ba ratios, the unreduced Sr/Ba ratio profiles had a higher density of data points across the profile length. To compare profiles of comparable data densities, Sr/Ba ratios were reduced by averaging adjacent values to obtain densities matching those of ^87^Sr/^86^Sr ratio profiles (approximately 1 measurement per 65 µm). Reduced Sr/Ba profiles were used for all subsequent analyses.

Isotope and elemental ratios from otolith edges should be representative of residence in the isolated waterholes prior to capture. Therefore, values from the ends of each profile reflecting the most recent months of growth (an average distance of 208±13 µm, 1 S.E.) were extracted and averaged over all fish collected from a given location. Differences in both ratios among locations were tested with non-parametric Kruskal-Wallis tests followed by multiple comparisons using the non-parameteric Dunn's test, which allows for unequal sample sizes [38]. Variances were calculated based on the number of fish in a given group.

Systematic shifts in values of both ratios across life history profiles were quantified using a global zoning algorithm [39]. This algorithm divides profiles into zones that are relatively chemically homogeneous and distinct from adjacent zones using a recursive process to identify shifts from one zone to another. For this study, we refer to these zones as habitats, as they ideally reflect movements between chemically distinct bodies of water. Zone breaks were pruned using a cross-validation approach repeated 1000 times to avoid model over-fitting. The kernel smoother window size was set to 0 µm, as profiles were already smoothed for the Sr/Ba ratios and at moderately low densities for ^87^Sr/^86^Sr ratios. Total numbers of chemically distinct habitats identified by the zoning algorithm for both ^87^Sr/^86^Sr and Sr/Ba ratio profiles were quantified and compared among individuals and by capture location.

## Results

**Figure 3:**
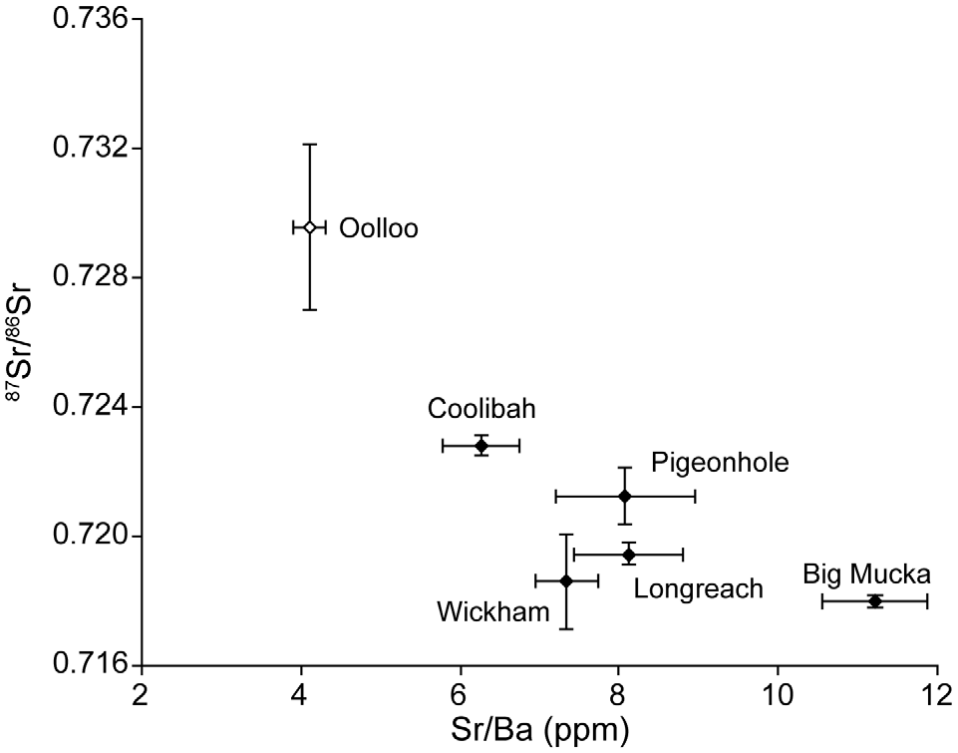
Edge otolith compositions. Isotope and trace element ratios from exterior edges of otoliths from Victoria River barramundi grouped by capture location.

Edge values corresponding to time spent in capture locations were significantly different for both ^87^Sr/^86^Sr (P<0.001) and Sr/Ba (P = 0.049) ratios. Non-parametric multiple comparisons revealed statistically significant differences between ratios for some pairs of sites but not others. In the case of ^87^Sr/^86^Sr ratios, Oolloo fish from the Daly River were significantly different from Big Mucka, Wickham, and Longreach fish. Fish from Big Mucka were also significantly different from Coolibah fish. In the case of Sr/Ba ratios, the only significant pairwise difference was between Oolloo and Big Mucka fish. Trends in isotope and elemental ratios across sites were observed (Figure 3). In general, lower Sr/Ba and higher ^87^Sr/^86^Sr ratios were found in fish from the most downstream Victoria River site (Coolibah) compared to those from upstream sites (e.g. Big Mucka). Fish from Oolloo in the Daly River had lower Sr/Ba and higher ^87^Sr/^86^Sr ratios compared to all Victoria River sites. Although not all of these sites were statistically significantly different from one another, these trends indicated detectable geological and chemical heterogeneity both between and within rivers.

**Figure 4:**
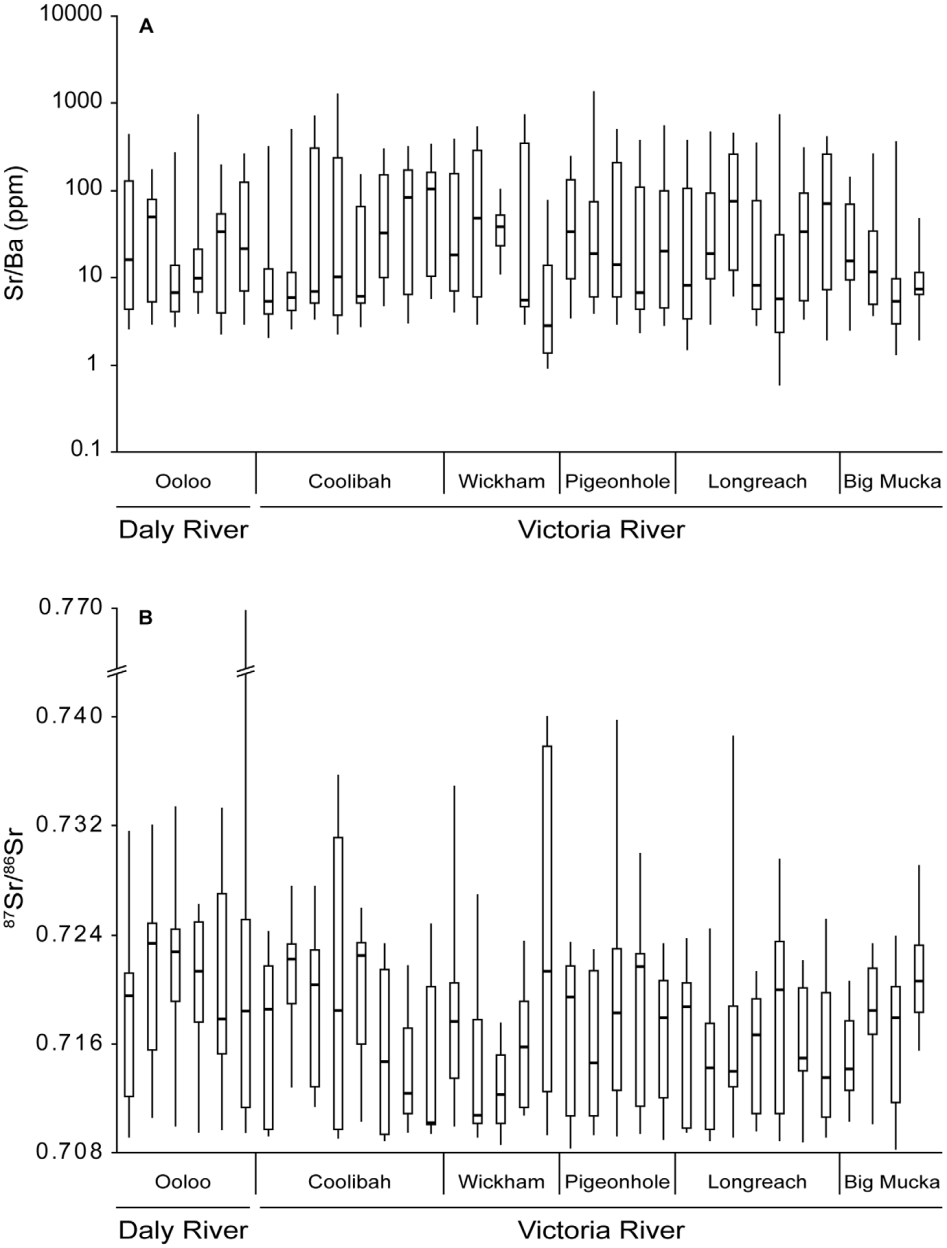
Boxplots of individual life history profiles. Boxplot summaries of otolith life history profiles grouped by capture location. Boxplots show (a) Sr/Ba and (b) ^87^Sr/^86^Sr ratios from individual profiles.

Individual life history profiles displayed high variability in both ^87^Sr/^86^Sr and Sr/Ba values (Figure 4, Figures S1, S2, S3, S4, S5, S6). Most profiles overlapped values typical of marine habitats for ^87^Sr/^86^Sr (0.70918) and Sr/Ba [approximately 50–1000; 21]. All individuals spent a significant amount of time in fresh water as well. Ranges for freshwater values of both ratios were high, indicating most fish moved across chemically heterogeneous habitats after emigrating from estuarine habitats. Many individuals recorded notably radiogenic (high) values of ^87^Sr/^86^Sr ratios, exceeding 0.730 in some cases and nearly reaching 0.770 in the case of one fish from the Daly River. Many of these values were outside the range constrained by those from otolith edges, suggesting fish movements over their lifetime were not restricted to those particular capture locations.

**Figure 5:**
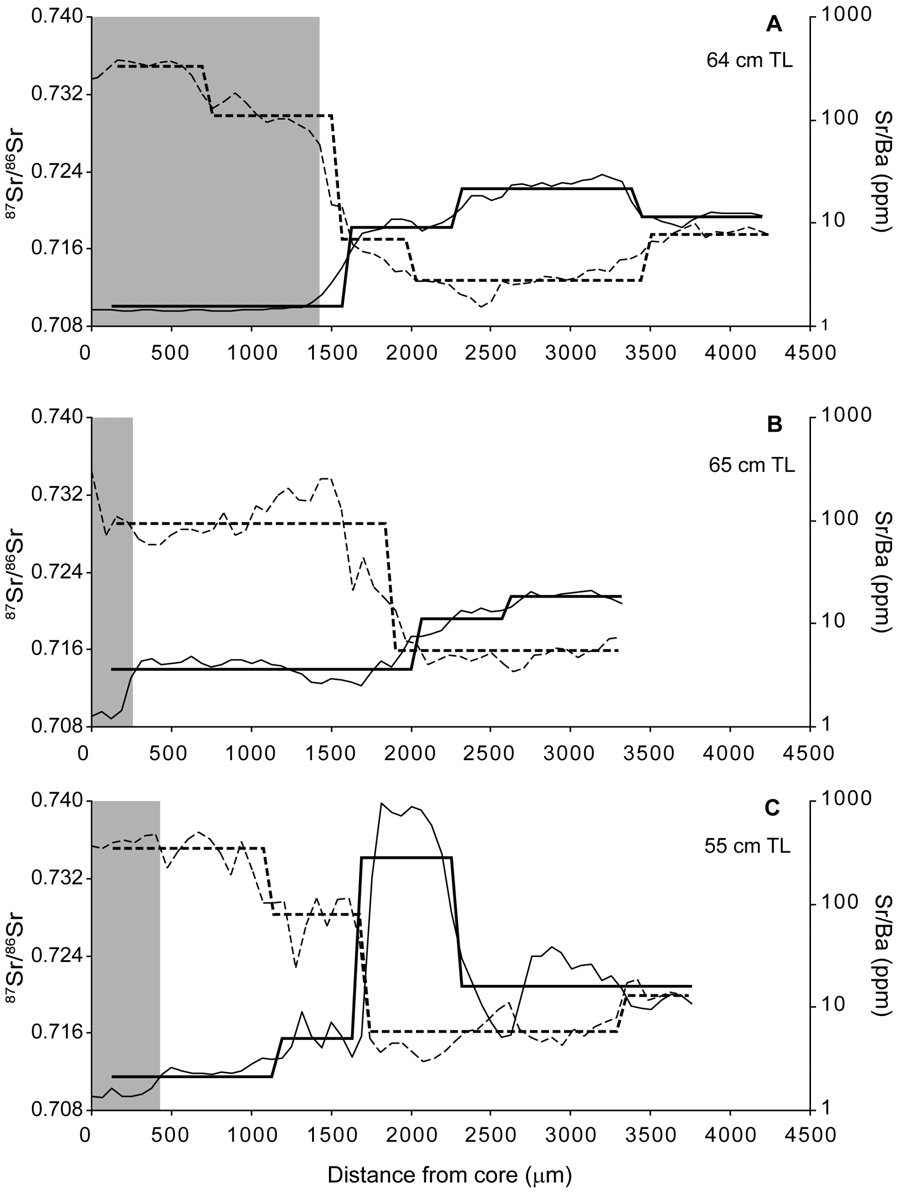
Representative life history profiles. Example life history profiles for three individual barramundi captured in the Victoria River. Profiles are shown from (a & c) Longreach and (b) Pigeonhole. Each profile shows values of Sr/Ba (dashed line) and ^87^Sr/^86^Sr ratios (solid line) from the core to the otolith edge. The shaded portion indicates marine residence. Fish total lengths (TL) are given in each panel.

A few distinct types of movement patterns were observed (Figure 5). Some individuals resided in fully marine habitats for an extended period before emigrating to fresh water (Figure 5a). In this case, Sr/Ba ratios indicated gradual movement across salinity gradients while ^87^Sr/^86^Sr ratios remained steady at marine values. Other profiles indicated rapid movement into estuarine and freshwater habitats, where they remained for the duration of their life (Figure 5b). Finally, movements between highly distinct freshwater chemical habitats were clear, and many individuals moved through high ^87^Sr/^86^Sr regimes after immigration into fresh water (Figure 5c).

**Figure 6:**
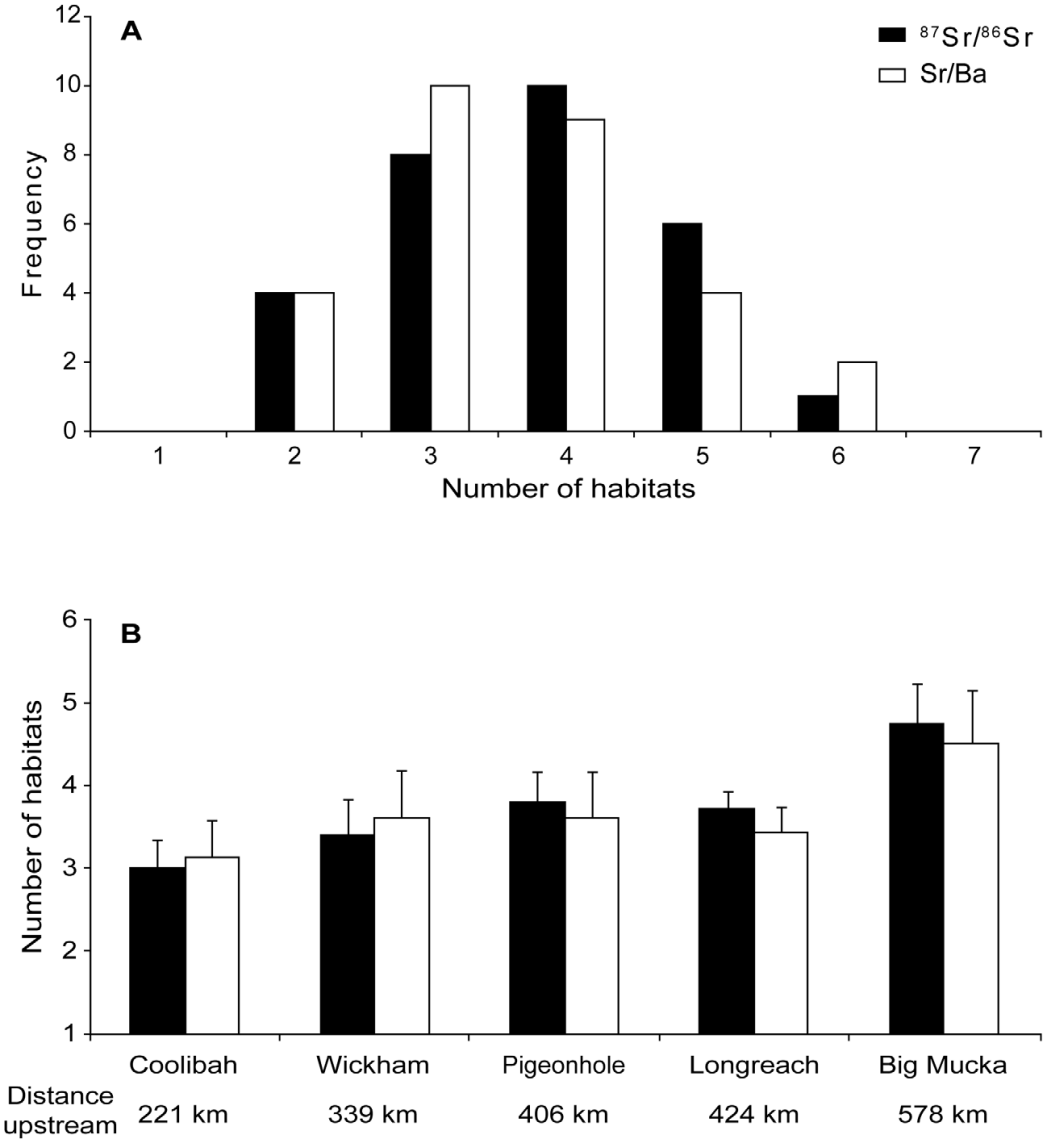
Distributions of habitat residency patterns. Summary distributions of habitat residency patterns. (a) Frequency distribution of the number of chemically distinct habitats recorded in each otolith life history profile from fish captured in the Victoria River. The number of habitats was defined using the global zoning algorithm of Hedger et al. [39]. (b) Mean (±1 SD) number of chemically distinct habitats in profiles from otoliths grouped by capture location. Results are shown for both ^87^Sr/^86^Sr (filled bars) and Sr/Ba ratios (open bars).

The zoning algorithm was effective at identifying large scale changes in values across ^87^Sr/^86^Sr and Sr/Ba ratio profiles. Short-term excursions were not detected, due to the smoothing nature of the algorithm. Estimates of the number of chemical habitats across profiles are therefore conservative, as they exclude short-term excursions to chemically distinct locations. Frequency distributions of the number of habitats identified by the zoning algorithm were similar for ^87^Sr/^86^Sr and Sr/Ba ratios (Figure 6a). The mean number of habitats identified in profiles from fish collected in the Victoria River was 3.6±0.2 (1 S.E.) for ^87^Sr/^86^Sr ratios and 3.6±0.2 for Sr/Ba ratios. Profile habitat numbers varied depending on capture location for Victoria River fish, with an increasing trend in the numbers of habitats identified in profiles from fish captured at the most downstream (Coolibah) to the furthest upstream location (Big Mucka; Figure 6b).

## Discussion

We detected significant heterogeneity in lifetime elemental and isotope ratios recorded in barramundi otoliths, indicating individually variable patterns of habitat use both across salinity gradients and within fresh water. Entry times into fresh water were variable, with some individuals moving quickly upstream and others lingering in estuarine or fully marine habitats. Once in fresh water, fish exhibited highly variable life history profiles, suggesting movement between distinct chemical regions within the rivers. These results indicate that fish make full use of multiple regions throughout a river when unimpeded by anthropogenic obstructions.

A central challenge for interpretations of otolith chemistry is identifying the source of variation in the selected elemental or isotope markers [40]. The two elements examined here, Sr and Ba, are thought to be primarily derived from water sources [11]–[12]. Although elemental incorporation rates can be modified by exogenous and endogenous factors including temperature, growth rate and physiological status [41]–[43], these effects are typically small in magnitude compared to those driven by the large gradients in dissolved ambient concentrations between fresh and marine habitats. Significant shifts in Sr/Ca and Ba/Ca ratios in barramundi otoliths were detected after experimental manipulation of water chemistry as well as transfer between fresh and marine waters [44]. Otolith ^87^Sr/^86^Sr ratios are also primarily determined by ambient values. Ratios of ^87^Sr/^86^Sr do not appear to fractionate trophically or during incorporation into biogenic structures [24], [45], although the routine normalization to specified ^88^Sr/^86^Sr values in order to account for instrumental fractionation also removes any natural mass dependent fractionation, if present [23], [46]. Regardless, otolith ^87^Sr/^86^Sr ratios have been repeatedly demonstrated to be effective recorders of ambient dissolved ^87^Sr/^86^Sr ratios making them powerful tags of residence in isotopically distinct habitats [16]–[17], [25], [47]. Thus, we are confident the chosen tracers for this study should be overwhelmingly driven by changes in water chemistry.

A related difficulty in interpreting otolith chemistry profiles is distinguishing between actual movement between chemically distinct locations and temporal fluctuations in ambient chemistry around stationary fish. Temporal fluctuations in water chemistry may be driven in part by seasonally variable stream flow. Flow-related alterations in ambient ^87^Sr/^86^Sr may be due to differential weathering of specific mineral types during various flow conditions, and the increased contribution of upstream values during high flow, provided those upstream tributaries are isotopically distinct. The evidence for flow-related variations in ^87^Sr/^86^Sr ratios is mixed. For instance, while some workers observed temporal stability in local dissolved freshwater ^87^Sr/^86^Sr ratios [48]–[50], others have reported significant relationships between water ^87^Sr/^86^Sr ratios and discharge rates [51]–[53]. These conflicting relationships are likely due to catchment–specific mineralogies, their spatial arrangement and flow dynamics. Solving this problem requires extensive spatial and temporal sampling of water or some other stationary biogenic proxy for local water chemistry in order to constrain estimates of seasonal and temporal variation in composition at a given location. Because of the remoteness of our study location, such a sampling effort was not feasible.

An alternative explanation for the patterns we observed is that the variations in otolith chemistry were the result of variable flow conditions. Although we cannot rule out the potential influence of variable flow rates on the otolith chemistry patterns we observed, several lines of evidence suggest that movement between river reaches is a more likely explanation. First, individual profiles were highly variable, with only some fish recording highly radiogenic ^87^Sr/^86^Sr ratios. If these values were the result of higher discharge altering downstream water chemistry around stationary fish, we would expect to see similar ranges in values for all individuals from a particular capture location. If this were the case, fish resident in a particular location would all experience the same fluctuations in environmental chemistry, regardless of their age. This would lead to comparable magnitudes of otolith chemical variation for all fish collected from a given location. Instead, we observed individually variable patterns regardless of their collection location. Second, discharge rates are highly seasonal, suggesting that flow-related chemical variation would be periodic and synchronous across individuals. Periodicity and synchronicity across otolith profiles was not observed. Third, following intuition, the highest number of habitats was identified for the most upstream sampling location (Big Mucka) with the lowest numbers of habitats identified for the sampling location furthest downsteam (Figure 6b). Fourth, previous tagging work has shown that barramundi can make extensive and rapid movements within rivers up to several kilometers per day in Northern Territory rivers [54]–[55]. These lines of evidence together suggest that the source of chemical variability across otoliths was predominantly due to individual movement patterns between chemically distinct habitats within different reaches of the catchments.

The highly radiogenic ^87^Sr/^86^Sr ratios recorded by some Victoria River fish were above the range of values recorded during the period of residence in any of the collection locations. Without a comprehensive set of water samples from all parts of the water course (650 km mainstem, >1200 km of tributaries), it is difficult to specifically pinpoint where fish might have moved to experience correspondingly high dissolved ^87^Sr/^86^Sr ratios. However, similar patterns of isotopic variability have also been observed in different formations of the nearby Ord River catchment, with values ranging from approximately 0.72 for Cambrian basalts up to between 0.74 to 0.79 for Proterozoic granites and associated weathered carbonates (M. McCulloch, unpublished data). Continental granitic rocks are typically enriched in Rb compared to carbonates, leading to relatively higher ^87^Sr/^86^Sr ratios in silicates due to decay of ^87^Rb to ^87^Sr over time [56]–[57]. Thus older bedrock terranes should contain on average the most radiogenic ratios in a region. Further, silicates typically contain broadly heterogeneous ^87^Sr/^86^Sr ratios at a variety of spatial scales, unlike carbonates such as limestones whose ^87^Sr/^86^Sr ratios tend to be relatively uniform in a given formation [58]. The upper reaches of the eastern portion of the Victoria River are dominated by Cambrian basalts, while the middle reaches and western tributaries span across Proterozoic sandstones, siltstones and shales. With the majority of the river composed of silicate formations, rock age may be a more significant variable in controlling the dissolved ^87^Sr/^86^Sr ratios. Because the western tributaries of the Victoria River are dominated by Proterozoic rocks, and none of our collection locations were in this region, those tributaries are likely candidates for habitats containing highly radiogenic dissolved ^87^Sr/^86^Sr ratios. This hypothesis is supported by the observation that the most radiogenic of the collection locations, Coolibah, was the only one surrounded by primarily Proterozoic rocks. If the dominance of Proterozoic rocks is indeed the source of the most radiogenic ^87^Sr/^86^Sr ratios, the barramundi otolith profiles indicate that some individuals made significant movements between the western and eastern branches of the Victoria River. Future work incorporating broad-scale water and fish sampling will be required to more tightly constrain the distribution of ^87^Sr/^86^Sr ratios throughout the river system.

Our results are consistent with previous research on barramundi movements prior to maturation in northern Australia. Juvenile barramundi primarily make use of tidal swamps and estuarine habitats before moving upstream into fresh water [59]–[63]. While fish in Papua New Guinea take up to three years to enter freshwater [64] and Queensland fish may not enter until four years of age, fish in the Van Diemen Gulf and the Gulf of Carpentaria were observed to enter freshwater within only one year [62]. Our data similarly suggest that fish in the Victoria and Daly rivers move rapidly into fresh water, although the precise timing of this migration appears individually variable. This limited time between spawning and fresh water entry promotes genetic structuring on a regional scale in northern Australia [65]–[66], although mixing among adjacent rivers may be facilitated by larval or juvenile transport by flood plumes [65]. The variability in fresh water entry times observed here is sufficient to support the notion that some individuals may inhabit marine environments long enough to allow such transport.

Importantly, our work quantified movements of immature individuals, indicating habitat use during pre-spawning periods. Further, for the Victoria River fish, these movements were quantified for individuals moving in unregulated systems. The ecological effects of river regulation are wide ranging and can significantly alter the structure and diversity of riverine communities [67]. Diadromous species worldwide have had to contend with severe reductions in available freshwater habitats either by anthropogenic barriers or extraction of surface water and groundwater [68]. For catadromous species like barramundi, this range reduction can restrict the available habitat for juveniles and pre- or post-spawning adults. Griffin [55] found that while mature males and females tended to reside in downstream brackish areas of the Daly River, immature males dominated farther upstream. This work, however, did not include sites upstream of Oolloo. In one of the few surveys of barramundi distributions in the upper reaches of NT catchments, Letnic and Connors [69] compiled historical and contemporary records and found barramundi distributions extended far inland in unobstructed systems. Our data similarly demonstrate that immature barramundi in both the Daly and Victoria rivers make extensive movements throughout the entire catchment.

Access to fresh water during immature life history stages is important for a variety of reasons. First, the productivity of freshwater habitats promotes higher growth of older juveniles and sub-adults [70]–[71]. Second, impoundments that restrict immature and adult fish to the lower reaches increases local densities and therefore competition for resources and cannibalism of juveniles [55]. Third, natural variation in flow rate itself cues the timing of migratory movements both upstream and downstream [62]. Barramundi are impeded by barrages even when fishways are provided for passage [72]. Unrestricted access to the extensive freshwater sections of river systems is therefore important for the persistence of barramundi populations. The otolith chemistry profiles from these unrestricted rivers in the Northern Territory demonstrate that variable movement patterns within fresh water habitats may represent life history plasticity that ensure robust barramundi populations in dynamic environments. The role of heterogeneous habitat use within fresh water should be integrated into theories that explore the effects of life history portfolios on the sustainability of diadromous species.

## Supporting Information

Figure S1 **Individual life history profiles from Oolloo.** Profiles are shown for all fish captured at Oolloo in the Daly River. Values of Sr/Ba (dashed line) and ^87^Sr/^86^Sr ratios (solid line) are shown from the core to the otolith edge. Fish total lengths (TL) are given in each panel. The ranges of some axes vary in order to accommodate the full range of individual data.
(TIF)

Figure S2 **Individual life history profiles from Coolibah.** Profiles are shown for all fish captured at Coolibah in the Victoria River, excluding those shown in Figure 5. Values of Sr/Ba (dashed line) and ^87^Sr/^86^Sr ratios (solid line) are shown from the core to the otolith edge. Fish total lengths (TL) are given in each panel. The ranges of some axes vary in order to accommodate the full range of individual data.
(TIF)

Figure S3 **Individual life history profiles from Wickham.** Profiles are shown for all fish captured at Wickham in the Victoria River. Values of Sr/Ba (dashed line) and ^87^Sr/^86^Sr ratios (solid line) are shown from the core to the otolith edge. Fish total lengths (TL) are given in each panel. The ranges of some axes vary in order to accommodate the full range of individual data.
(TIF)

Figure S4 **Individual life history profiles from Pigeonhole**. Profiles are shown for all fish captured at Pigeonhole in the Victoria River, excluding those shown in Figure 5. Values of Sr/Ba (dashed line) and ^87^Sr/^86^Sr ratios (solid line) are shown from the core to the otolith edge. Fish total lengths (TL) are given in each panel. The ranges of some axes vary in order to accommodate the full range of individual data.
(TIF)

Figure S5 **Individual life history profiles from Longreach.** Profiles are shown for all fish captured at Longreach in the Victoria River, excluding those shown in Figure 5. Values of Sr/Ba (dashed line) and ^87^Sr/^86^Sr ratios (solid line) are shown from the core to the otolith edge. Fish total lengths (TL) are given in each panel. The ranges of some axes vary in order to accommodate the full range of individual data.
(TIF)

Figure S6 **Individual life history profiles from Big Mucka.** Profiles are shown for all fish captured at Coolibah in the Victoria River. Values of Sr/Ba (dashed line) and ^87^Sr/^86^Sr ratios (solid line) are shown from the core to the otolith edge. Fish total lengths (TL) are given in each panel. The ranges of some axes vary in order to accommodate the full range of individual data.
(TIF)
