# Supplementary figures and images for: Movements of Diadromous Fish in Large Unregulated Tropical Rivers Inferred from Geochemical Tracers
Source: PLoS One. 2011 Apr 6;6(4):e18351. doi: 10.1371/journal.pone.0018351 (PMC3071836; doi:10.1371/journal.pone.0018351)

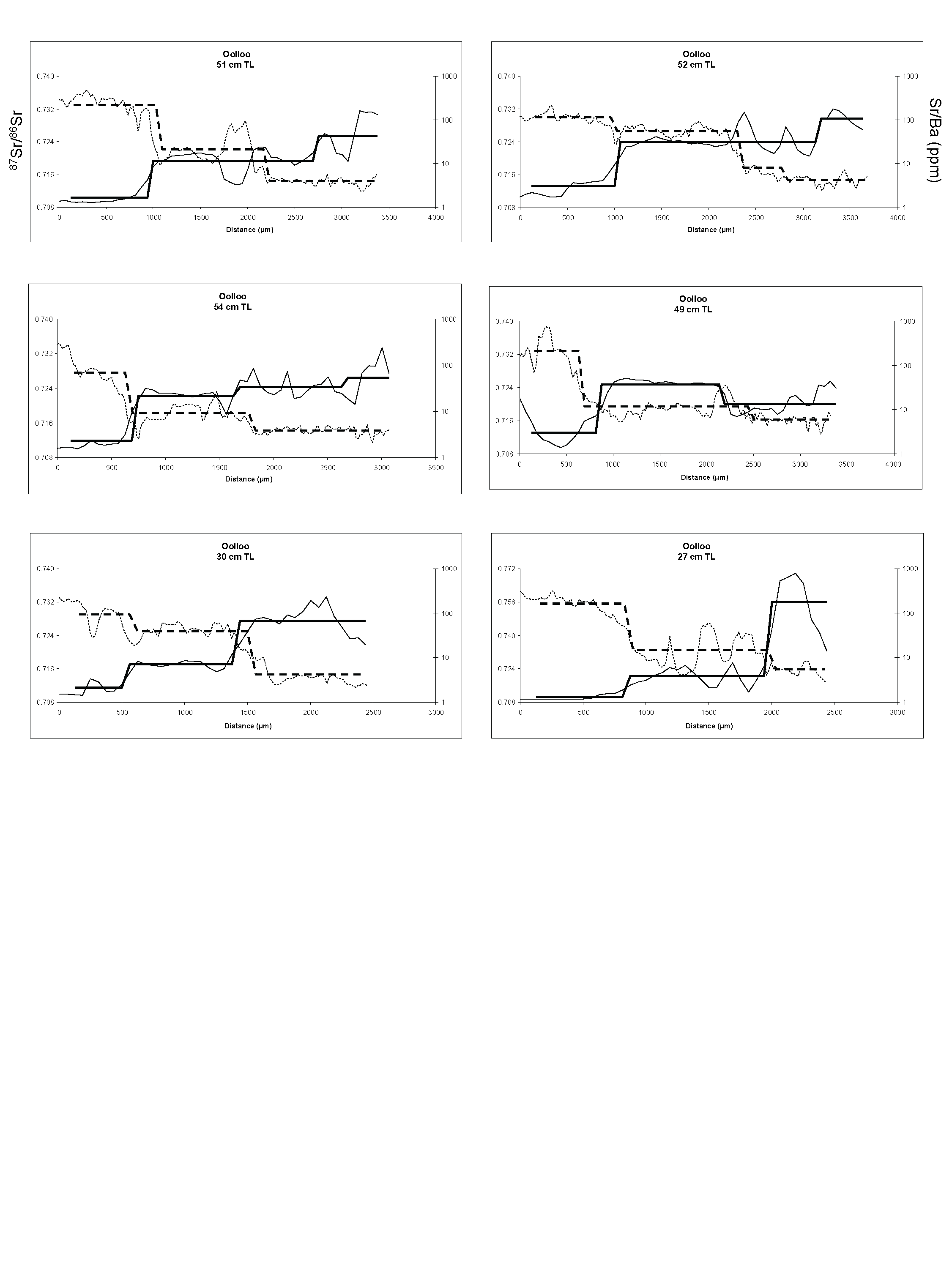

Supplement: Figure S1 — Individual life history profiles from Oolloo. Profiles are shown for all fish captured at Oolloo in the Daly River. Values of Sr/Ba (dashed line) and 87Sr/86Sr ratios (solid line) are shown from the core to the otolith edge. Fish total lengths (TL) are given in each panel. The ranges of some axes vary in order to accommodate the full range of individual data. (TIF) [file pone.0018351.s001.tif]

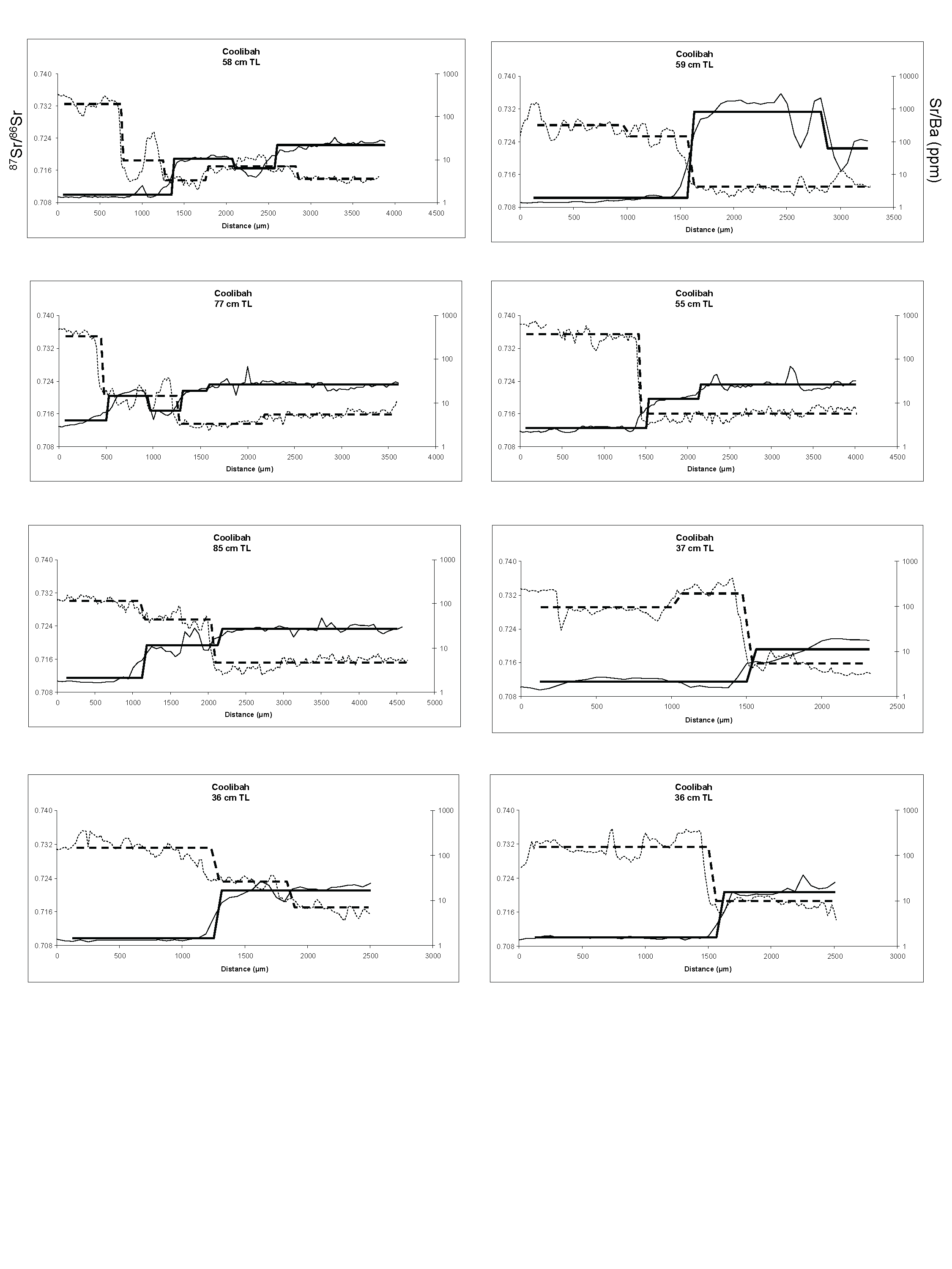

Supplement: Figure S2 — Individual life history profiles from Coolibah. Profiles are shown for all fish captured at Coolibah in the Victoria River, excluding those shown in Figure 5. Values of Sr/Ba (dashed line) and 87Sr/86Sr ratios (solid line) are shown from the core to the otolith edge. Fish total lengths (TL) are given in each panel. The ranges of some axes vary in order to accommodate the full range of individual data. (TIF) [file pone.0018351.s002.tif]

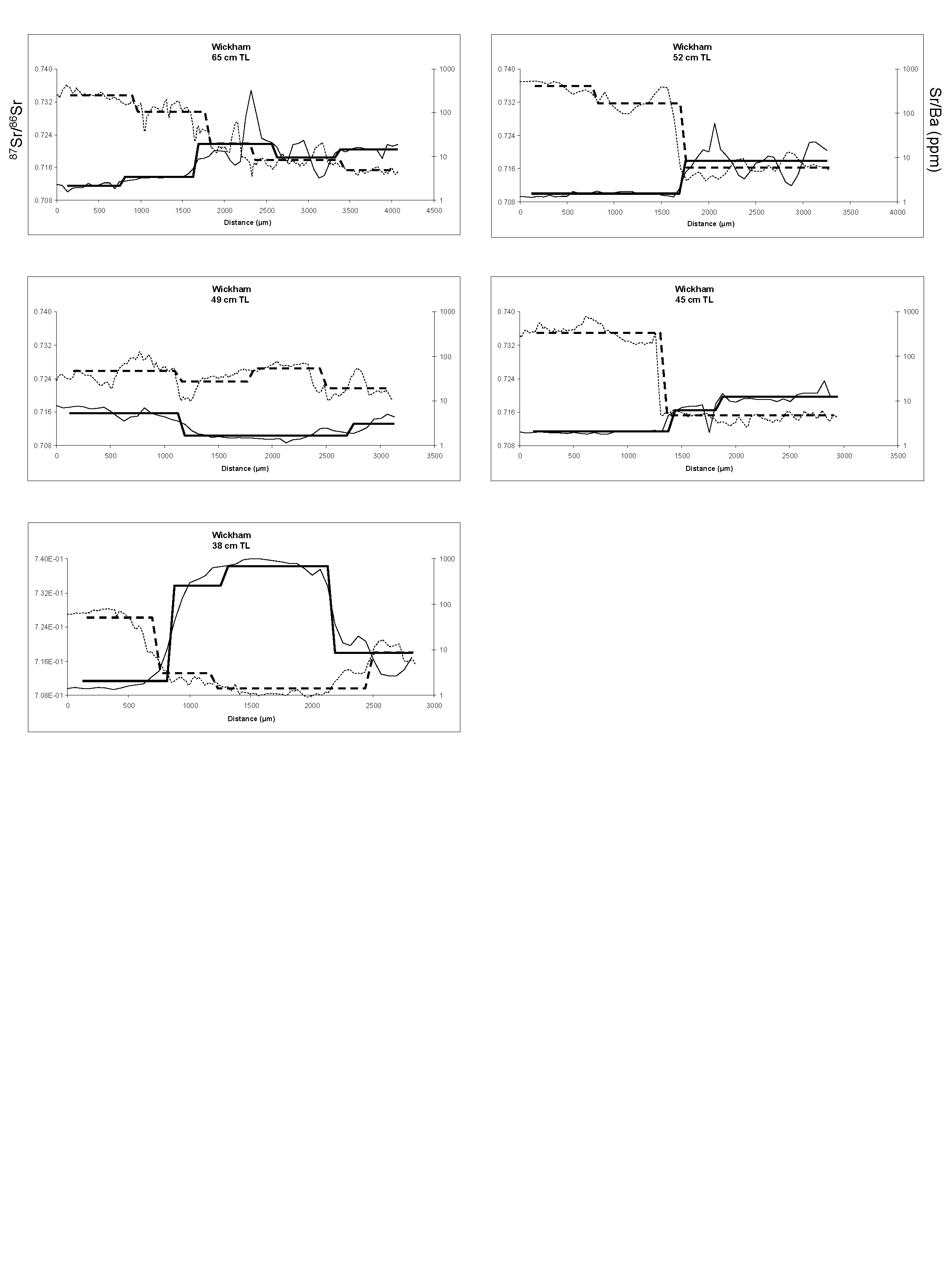

Supplement: Figure S3 — Individual life history profiles from Wickham. Profiles are shown for all fish captured at Wickham in the Victoria River. Values of Sr/Ba (dashed line) and 87Sr/86Sr ratios (solid line) are shown from the core to the otolith edge. Fish total lengths (TL) are given in each panel. The ranges of some axes vary in order to accommodate the full range of individual data. (TIF) [file pone.0018351.s003.tif]

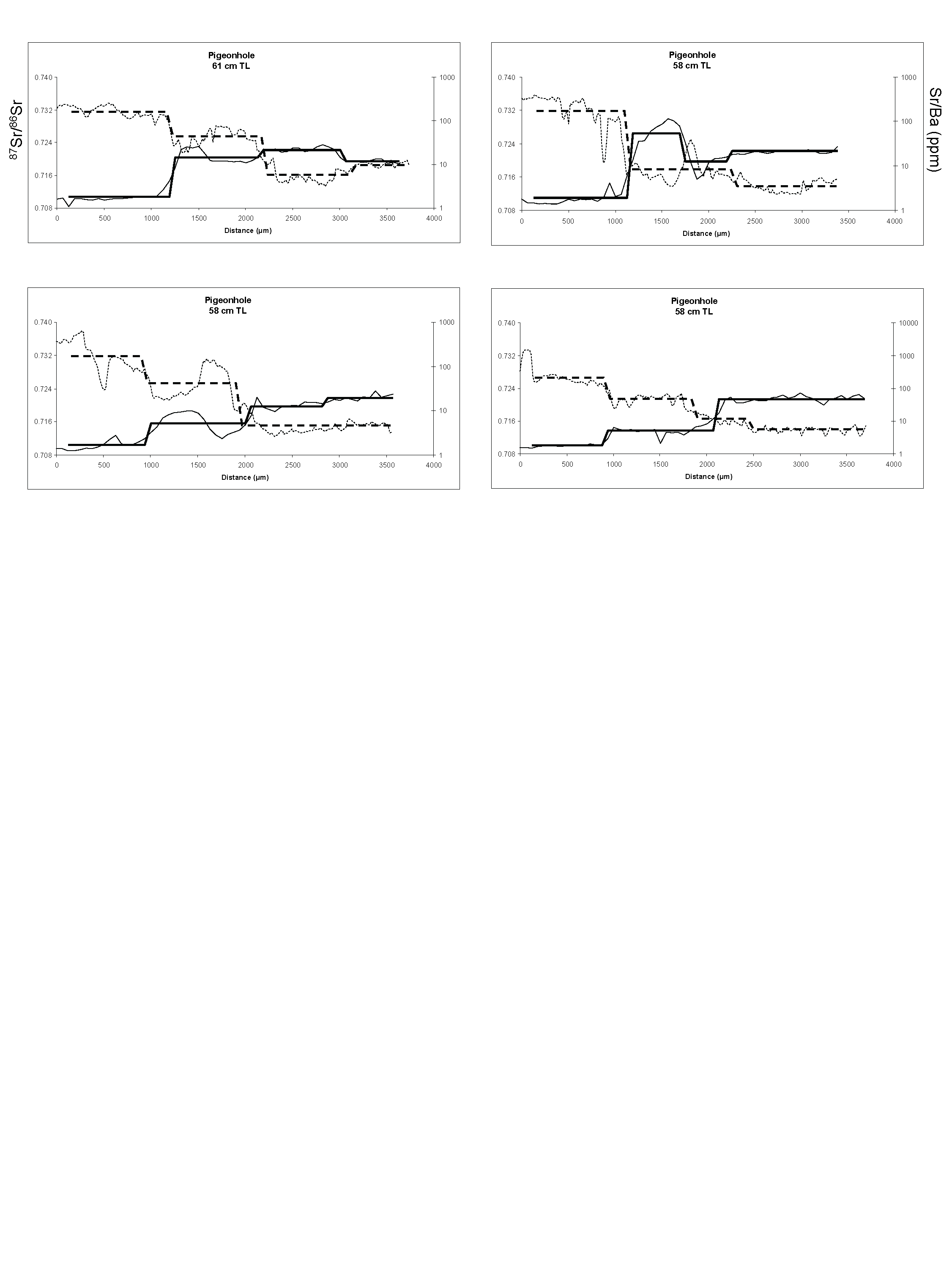

Supplement: Figure S4 — Individual life history profiles from Pigeonhole. Profiles are shown for all fish captured at Pigeonhole in the Victoria River, excluding those shown in Figure 5. Values of Sr/Ba (dashed line) and 87Sr/86Sr ratios (solid line) are shown from the core to the otolith edge. Fish total lengths (TL) are given in each panel. The ranges of some axes vary in order to accommodate the full range of individual data. (TIF) [file pone.0018351.s004.tif]

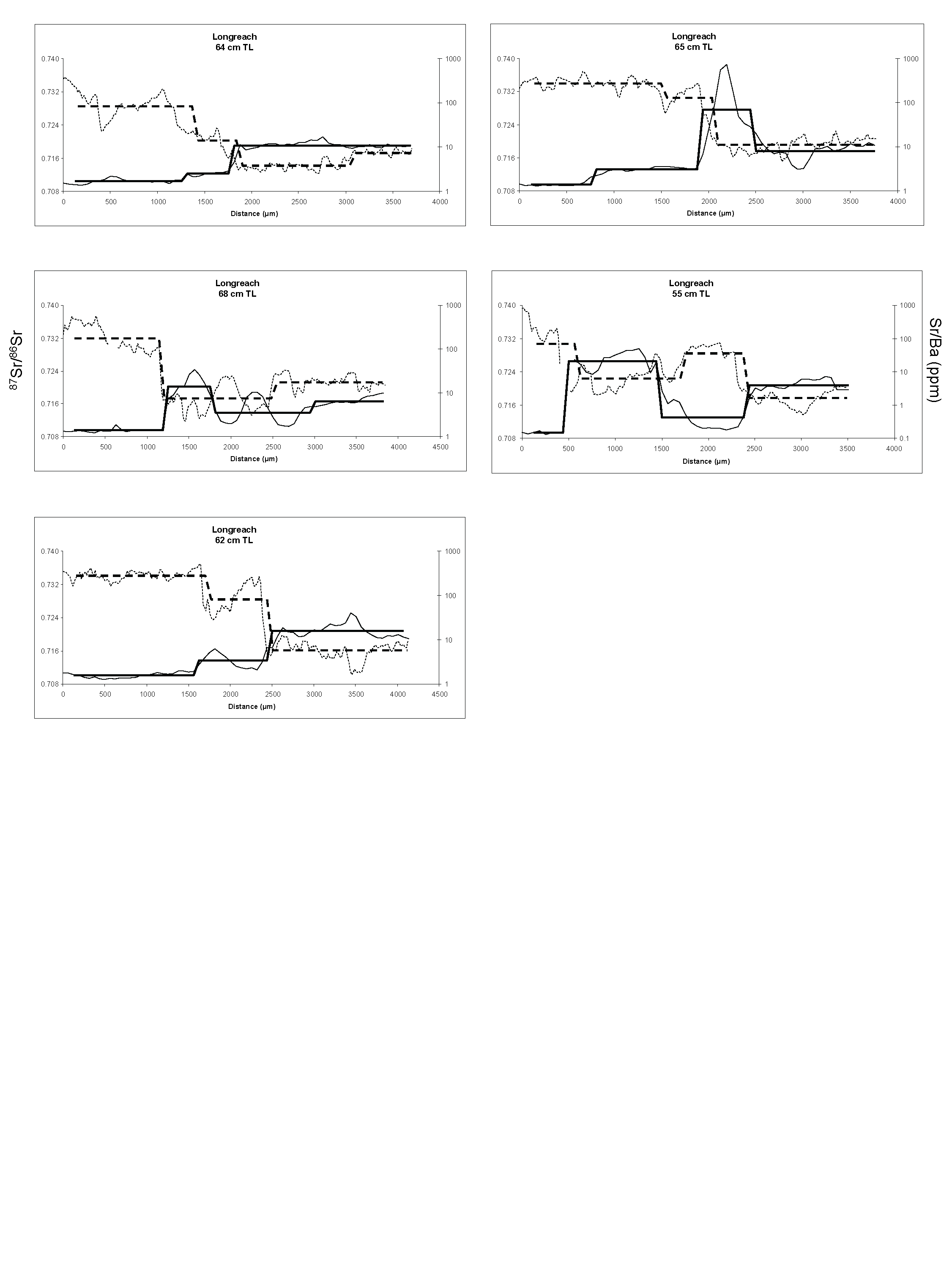

Supplement: Figure S5 — Individual life history profiles from Longreach. Profiles are shown for all fish captured at Longreach in the Victoria River, excluding those shown in Figure 5. Values of Sr/Ba (dashed line) and 87Sr/86Sr ratios (solid line) are shown from the core to the otolith edge. Fish total lengths (TL) are given in each panel. The ranges of some axes vary in order to accommodate the full range of individual data. (TIF) [file pone.0018351.s005.tif]

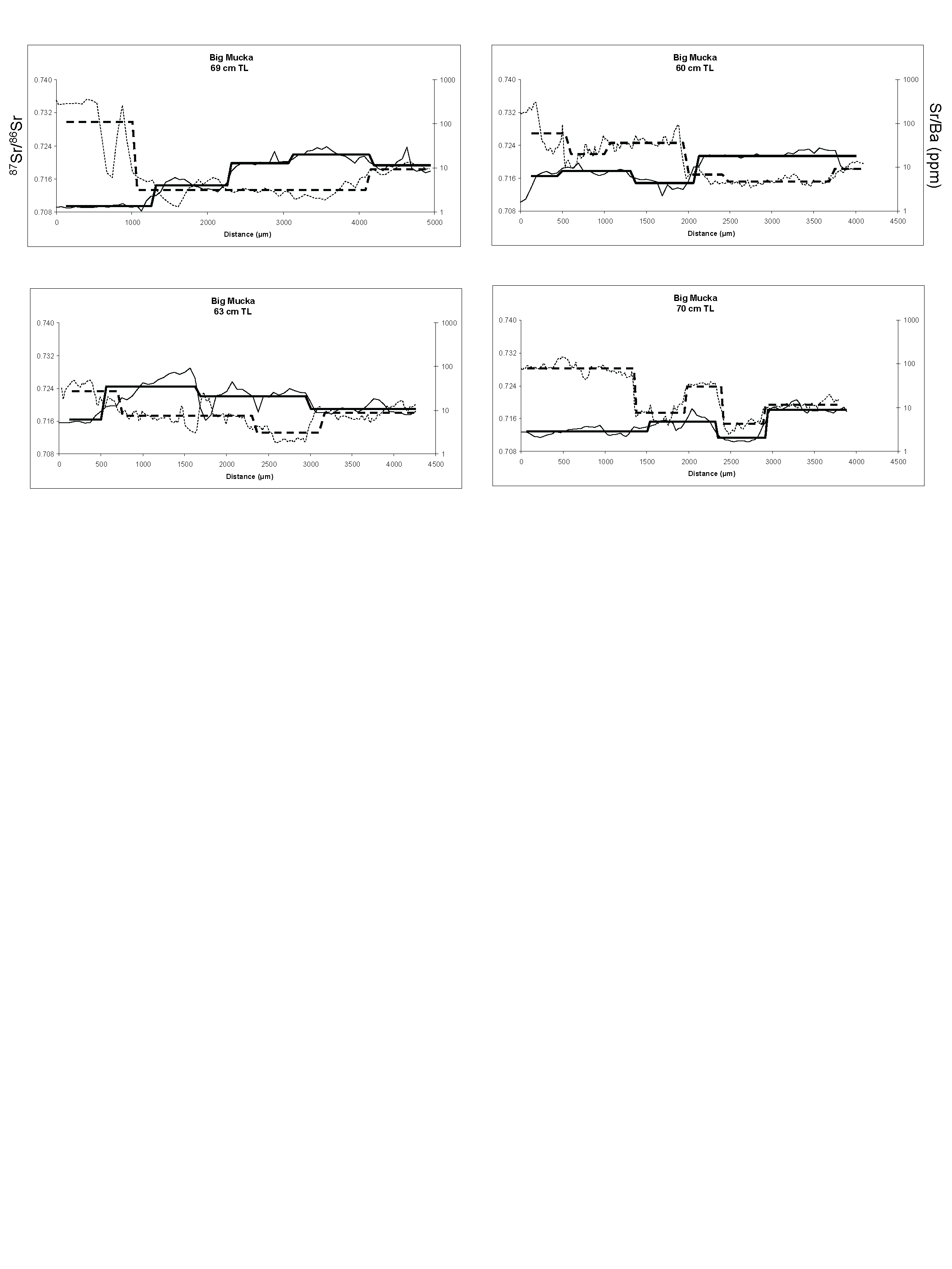

Supplement: Figure S6 — Individual life history profiles from Big Mucka. Profiles are shown for all fish captured at Coolibah in the Victoria River. Values of Sr/Ba (dashed line) and 87Sr/86Sr ratios (solid line) are shown from the core to the otolith edge. Fish total lengths (TL) are given in each panel. The ranges of some axes vary in order to accommodate the full range of individual data. (TIF) [file pone.0018351.s006.tif]
